# In Vitro Modulation of Endogenous Antioxidant Enzyme Activities and Oxidative Stress in Autism Lymphoblastoid Cell Line (ALCL) by Stingless Bee Honey Treatment

**DOI:** 10.1155/2020/4539891

**Published:** 2020-12-01

**Authors:** Nazatul Shima Nayan, Muhammad Aiman Mohd Yazid, Kamachisree Nallappan, Amirul Asyiq Amran, Nur Syuhaidah Zaidi, Fazaine Zakaria, Hazirah Hassan, Norwahidah Abdul Karim

**Affiliations:** Department of Biochemistry, Faculty of Medicine, Universiti Kebangsaan Malaysia Medical Centre, 56000 Cheras, Kuala Lumpur, Malaysia

## Abstract

Autism has been associated with a low antioxidant defense mechanism, while honey has been known for decades for its antioxidant and healing properties. Determination of stingless bee honey (KH) effects on antioxidant enzyme activities and oxidative damage in Autism Lymphoblastoid Cell Line (ALCL) was performed. ALCL and its normal sibling pair (NALCL) were cultured in RPMI-1640 medium at 37°C and 5% CO_2_. ALCL was treated with 400 *μ*g/mL KH (24 h), and oxidative stress marker, malondialdehyde (MDA), and antioxidant enzyme activities (catalase (CAT), glutathione peroxidase (GPx), and superoxide dismutase (SOD)) were measured via enzyme-linked immunosorbent assay (ELISA), while deoxyribonucleic acid (DNA) damage was determined via comet assay. Low SOD activity (*p* < 0.05) and high MDA level (*p* < 0.05) were observed in ALCL compared to NALCL. Higher grade (Grades 2 and 3) of DNA damage was highly observed (*p* < 0.05) in ALCL compared to NALCL, whereas lower grade (Grades 0 and 1) DNA damage was highly detected (*p* < 0.05) in NALCL compared to ALCL. KH treatment caused a significant increase in SOD and GPx activities (*p* < 0.05) in ALCL compared to untreated ALCL. Correspondingly, KH treatment reduced the Grade 2 DNA damage (*p* < 0.05) in ALCL compared to untreated ALCL. CAT activity showed no significant difference between all three groups, while the MDA level showed no significant difference between treated and untreated ALCL. In conclusion, KH treatment significantly reduced the oxidative stress in ALCL by increasing the SOD and GPx antioxidant enzyme activities, while reducing the DNA damage.

## 1. Introduction

Autism spectrum disorder (ASD), also recognized as autism, is an extremely heritable disorder. It is also known as a heterogeneous neurodevelopmental disorder with impaired social, verbal, or nonverbal interaction; sensory anomalies; stereotyped and repetitive behaviors or interests; and varying levels of intellectual disability [1]. Medical comorbidities such as epilepsy, sleep disturbances, gastrointestinal dysfunction, immune dysfunction, and mental retardation may accompany the core behavioral abnormalities mentioned [2].

The prevalence of ASD was estimated to be 1 in 160 children worldwide [3]. Up to the present, various aspects from environmental factors to epigenetic perspective and immunological relativity had been linked to autism. However, no valid determinant had been approved as the underlying cause of ASD [4]. Excessive oxidative stress and antioxidant defense mechanism impairment had been correlated with autism [4]. Oxidative damages triggered by the imbalance of the excessive production of reactive oxygen species and weaken antioxidant defense may contribute to the pathogenesis of the disorder and clinical signs and symptoms [4].

Regulation of redox balance is the main focus of current reports on autism and antioxidant therapy as the main player. Clinical trials had been carried out by using various kinds of antioxidants such as vitamin C, glutathione, fish oil, melatonin, carnosine and zinc, and vitamin B6-magnesium combination [4]. It was reported that the treatment with high doses of vitamin C improved the behavioral symptoms of autism [5]. Other than antioxidants, nutraceutical supplementation on ASD individuals such as dietary fatty acids, herbal preparation, and probiotics also showed convincing outcomes on reducing the progress of ASD [6].

The correlation of redox imbalance and potential redox therapy with ASD pathogenesis inspired the interest in studying *kelulut* honey (KH), a natural product, on autism cell lines. Widely considered as having antioxidant properties, the total antioxidant activity of honey is contributed by its complex chemical components such as phenols, flavonoids, phenolic acids, proteins, amino acids, and vitamins [7]. Honey is also reported to exhibit other therapeutic activities such as antimicrobial, anti-inflammatory, antineoplastic, wound healing, and protective effects on the liver and cardiovascular system [8]. In this study, the modulatory effect of KH antioxidant properties on oxidative stress levels in Autism Lymphoblastoid Cell Line (ALCL) was determined by measuring the endogenous antioxidant enzyme activities and DNA damages in cells.

*Trigona* spp. is the bee species involved in the production of KH. Derived from multiflora sources, KH was reported to be ten times higher in antioxidant properties [9, 10]. Reports from the previous studies showed that the total phenolic content (TPC) of the KH was higher compared to New Zealand manuka honey [9, 10] and the total flavonoid content (TFC) was higher from Portuguese heather honey and Spanish rosemary honey [10, 11]. Additionally, ferric-reducing antioxidant potential (FRAP) assay showed that the antioxidant activity of KH was higher than that was found in New Zealand manuka honey [12].

## 2. Materials and Methods

### 2.1. KH Sample

The KH sample was purchased from Persatuan Usahawan Lebah Kelulut Darul Naim (DRONESS), Kelantan, Malaysia. KH was produced by *Trigona* spp. (a stingless bee species) and produced from a multiflora source. The honey was obtained from Kelantan, Malaysia, where the beekeepers are the community of the DRONESS. The optimum dose of KH at 400 *μ*g/mL was obtained from the previous viability assay (unpublished data) and used as the treatment on ALCL. The honey sample was prepared in the culture medium on the day of the assay.

### 2.2. Cell Culture

Three variants of Autism Lymphoblastoid Cell Line (ALCL) were used as the autism model and its nonaffected siblings (NALCL) for the normal control. Cell lines were provided by the “Autism Genetic Resource Exchange” (AGRE; Los Angeles, CA, USA). The cell lines were cultured in the “Roswell Park Memorial Institute” (RPMI) 1640 media (Sigma-Aldrich, German) supplemented with 15% (*v*/*v*) fetal bovine serum (FBS; Sigma-Aldrich, German), 2 mM L-glutamine (Gibco, USA), 100 U mL^−1^ penicillin, and 100 U mL^−1^ streptomycin (Gibco, USA) at 37°C and 5% CO_2_. The cell lines used were from the 8^th^ to the 10^th^ passage. The cells were divided into three groups: ALCL, KH-treated ALCL, and NALCL. Oxidative stress marker such as malondialdehyde (MDA) and antioxidant enzyme activities such as catalase (CAT), glutathione peroxidase (GPx), and superoxide dismutase (SOD) were measured using specific “enzyme-linked immunosorbent assay (ELISA) kits” (Cayman Chemical, USA) while deoxyribonucleic acid (DNA) damage was measured using “comet assay” (Sigma-Aldrich, USA). All methods mentioned below were based on the manufacturer's protocols.

### 2.3. Superoxide Dismutase (SOD) Assay

Cells were collected by 10 min centrifugation (1,000–2,000 × *g*) (4°C). The cell pellet was homogenized and sonicated in cold 20 mM HEPES buffer (pH 7.2) (70 mM sucrose, 210 mM mannitol, and 1 mM EGTA). It was then centrifuged at 1,500 × *g* (5 min) (4°C). The supernatant was used for the analysis.

The reactions were started by adding 20 *μ*L xanthine oxidase (diluted) to the samples. The procedure was done according to the manufacturer's protocols. The readings were recorded at 440–460 nm.

### 2.4. Glutathione Peroxidase (GPx) Assay

Cells were collected by centrifugation (1,000–2,000 × *g*) (10 min) (4°C). Pellet was homogenized in a cold buffer and centrifuged at 10,000 × *g* (15 min) (4°C). The supernatant was used for the analysis.

The samples were concentrated using an Amicon centrifuge concentrator to achieve the optimum enzymatic activity. The reactions were started by adding 20 *μ*L of cumene hydroperoxide to the samples. The procedure was done according to the manufacturer's protocols. The readings were recorded at 340 nm (five time points).

### 2.5. Catalase (CAT) Assay

Cells were collected by centrifugation (1,000–2,000 × *g*) (10 min) (4°C), homogenized and sonicated on ice in 1–2 mL of cold buffer. It was then centrifuged (10,000 × *g*) (15 min) (4°C). The supernatant was used for analysis.

The reactions were started by adding H_2_O_2_ to the samples. The procedure was done according to the manufacturer's protocols. The readings were recorded at 540 nm.

### 2.6. Malondialdehyde (MDA) Assay

2 × 10^7^ cells were collected in 1 mL of cell culture media. The cells were homogenized and sonicated on ice, and the whole homogenate was used in the assay. Cell lysates do not need to be diluted before assaying.

100 *μ*L of sample and 100 *μ*L SDS solution were added to 5 mL vial. 4 mL of the color reagent was added to the sides of vials and placed on a floating foam tube holder. The vials were added to boiling water (1 h) followed by an ice bath (10 min). The procedure was done according to the manufacturer's protocols. The readings were recorded at 530–540 nm.

### 2.7. Comet Assay

Comet assay was done to determine DNA damage using “single-cell gel electrophoresis” (SCGE). Slides were covered with 0.6% of “Normal Melting Agarose” (NMA), a mixture of cell suspension, 0.6% of “Low Melting Agarose” (LMA), and 0.6% of LMA (without cell). The solidification process was done at 4°C. Slides were then immersed in the “lysing buffer” (2.6 M NaCl, 100 mM Na_2_EDTA, 10 mM Tris, and 1% Triton-X, pH 10) (4°C) (1 h).

Before electrophoresis, the slides were left in the electrophoresis solution (pH 13) (20 min). Electrophoresis was done at 4°C (20 min) (25 V and 0.3 A). Finally, the slides were neutralized, stained with ethidium bromide, and analyzed using a fluorescent microscope. The degree of DNA damage is graded into five groups based on the amount of the DNA in the tail (Table 1).

### 2.8. Statistical Analysis

All statistical analyses were performed using SPSS version 23.0 with statistical significance set at *p* < 0.05. Differences between the variables between ALCL, KH-treated ALCL, and NALCL were compared using one-way ANOVA. Three variants of ALCL were used, and all assays were performed in triplicate, with 24 h incubation time of KH treatment.

## 3. Results

### 3.1. Endogenous Antioxidant Enzyme Activities in ALCL and KH Treatment

**Figure 1:**
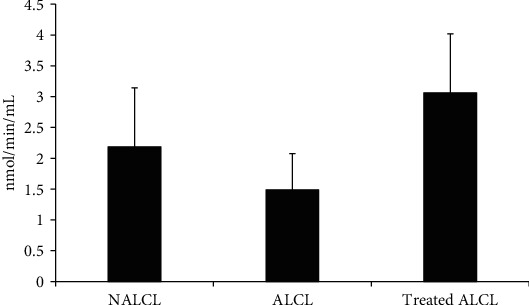
CAT activities in different groups of cells, normal (NALCL), autism (ALCL), and KH-treated ALCL. Results are expressed as mean ± SD.

CAT activity showed no significant difference between the normal group of cell lines (NALCL) and the autism group (ALCL; *p* > 0.05; Figure 1). KH treatment on ALCL for 24 h incubation time showed no significant difference with untreated ALCL (*p* > 0.05). All three groups of cell lines showed no significant difference in CAT activity (*p* > 0.05).

**Figure 2:**
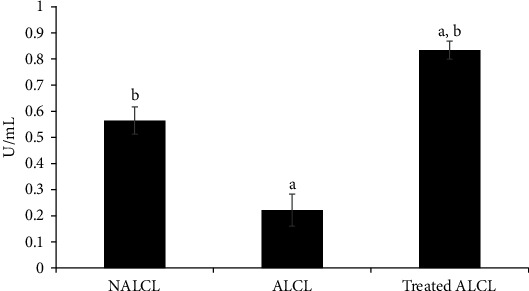
SOD activities in different groups of cells, normal (NALCL), autism (ALCL), and KH-treated ALCL. Results are expressed as mean ± SD. ^a^Denotes *p* < 0.05 compared to NALCL. ^b^Denotes *p* < 0.05 compared to ALCL.

SOD activity was found to be significantly lower in the autism cell lines (ALCL) compared to NALCL (*p* < 0.05; Figure 2). Interestingly, the SOD activity was successfully restored and significantly increased with 24 h KH treatment relative to the untreated ALCL and NALCL groups (*p* < 0.05).

**Figure 3:**
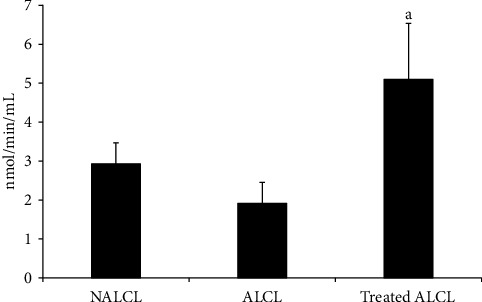
GPx activities in different groups of cells, normal (NALCL), autism (ALCL), and KH-treated ALCL. Results are expressed as mean ± SD. ^a^Denotes *p* < 0.05 compared to ALCL.

GPx activity in NALCL did not significantly differ from the ALCL group (*p* > 0.05; Figure 3). On the contrary, the KH-treated ALCL group showed a significant increase in GPx activity relative to untreated ALCL with 24 h treatment (*p* < 0.05).

### 3.2. Oxidative Damages in ALCL and KH Treatment

**Figure 4:**
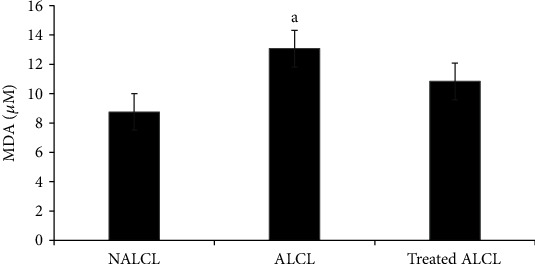
MDA concentrations in different groups of cells, normal (NALCL), autism (ALCL), and KH-treated ALCL. Results are expressed as mean ± SD. ^a^Denotes *p* < 0.05 compared to NALCL.

MDA concentrations were found to be significantly higher in the autism group (ALCL) than the normal group, NALCL (*p* < 0.05; Figure 4). However, MDA concentration in treated ALCL did not significantly decrease with KH treatment compared to the ALCL and NALCL groups (*p* > 0.05).

**Figure 5:**
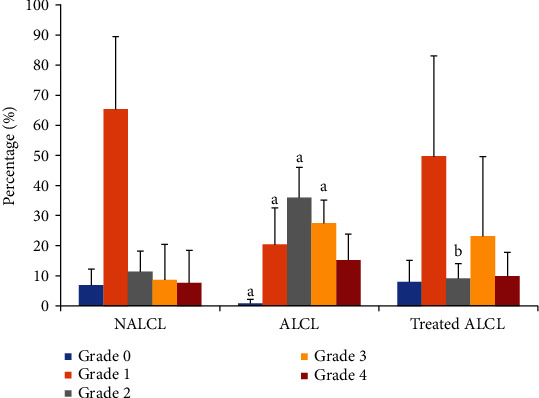
Percentage of DNA damage in different groups of cells, normal (NALCL), autism (ALCL), and KH-treated ALCL. Results are expressed as mean ± SD. ^a^Denotes *p* < 0.05 compared to NALCL. ^b^Denotes *p* < 0.05 compared to ALCL.

Figure 5 represents the outcomes of DNA damage and demonstrates that Grade 0 (no DNA damage) and Grade 1 (low-level DNA damage) are significantly higher in the normal cell lines (NALCL) compared to the autism cell lines, ALCL (*p* < 0.05). However, Grade 2 (medium-level DNA damage) and Grade 3 (high-level DNA damage) are significantly higher in ALCL (*p* < 0.05). Interestingly, the 24 h KH treatment on ALCL significantly reduced the Grade 2 DNA damage compared to the untreated group (*p* < 0.05).

## 4. Discussion

### 4.1. Modulation of Endogenous Antioxidant Enzyme Activities in ALCL by KH

SOD, CAT, and GPx play a vital role in protecting cells from oxidative stress [4]. They are the three antioxidant enzymes measured in this study. During the oxidative chain, superoxide is the primary ROS produced and will be further converted to hydrogen peroxide (H_2_O_2_) by SOD and to water by CAT and/or GPx activities [4]. The vital functions of endogenous antioxidant defense are to halt the ROS production and deactivate the propagated ROS [4]. Oxidative stress is the consequence of the imbalance between the uncontrolled production of ROS and the compromised defense mechanism which eventually caused oxidative damages [4].

Previous studies have reported that the CAT activity is lower in erythrocytes of autistic patients. Other studies showed the reduction of the total GSH levels and altered GPx, SOD, and CAT activities in patients with autism compared to the controls [4]. In this study, the three vital antioxidant enzymes CAT, SOD, and GPx were measured to determine the differences of activities in three groups of cell lines. The findings that showed no significant difference in CAT activities between the ALCL and NALCL groups were in line with the other findings from a study conducted on Saudi autistic children [13]. Nonetheless, KH treatment failed to enhance the activity of CAT in ALCL.

A study showed that a higher level of H_2_O_2_ (a type of ROS) was generated in ASD children, as indicated by the decrease of superoxide dismutase (SOD) and glutathione peroxidase (GPx) activities in erythrocytes [14]. Our data revealed that the activity of SOD was significantly lower in ALCL compared to NALCL. Interestingly, the SOD activity could be restored and significantly increased with KH treatment. The ability of KH to regenerate the SOD activity in the autism cell lines could be contributed by the antioxidant capacity of the honey. Reports from other studies using an antioxidant source of treatments such as boiled camel milk [15] and vitamin C and E [16] similarly showed a significant increase in SOD activity in ASD and sickle cell patients, respectively. Likewise, hesperetin and nanohesperetin (antioxidant) treatments also showed enhanced SOD expression and ameliorated social behavior deficits in autistic Wistar-Albino rats [17].

Based on the previous studies, autistic patients showed a significantly lower plasma GPx than controls [18, 19]. On the contrary, Thomas et al. reported that the GPx activity in sickle cell patients was significantly increased after they received vitamin C and E antioxidant treatments [16]. Camel milk and hesperetin intervention also showed significant increment levels in GPx and enhanced GPx expression in autistic patients and autistic Wistar-Albino rats, besides improving the autistic behavior [15, 17]. Similar findings were determined in this study, where GPx activity was found to be significantly higher after 24 h KH treatment in the autism cell lines, ALCL. The substantial increase in GPx and SOD activities in the treated autism cell lines may indicate the capacity of KH in modulating and restoring the activity of the endogenous antioxidant enzymes in autism cell lines.

### 4.2. Oxidative Stress and the Reduction of DNA Damage following KH Treatment in ALCL

Lipids especially polyunsaturated fatty acids (PUFA) are oxidized by ROS which further initiating oxidative damage [4]. Malondialdehyde (MDA) is the product of lipid oxidation that has been established as an oxidative damage marker [4]. Numerous studies have indicated increased oxidative stress levels in individuals with ASD [20]. In addition to the elevation of serum lipid peroxides, the markers of lipid peroxidation, and urinary isoprostane levels [20], elevated levels of cytokines and xanthine oxidase have also been noted in the blood circulation of autistic patients, both of which generate ROS [20].

MDA concentration has been reported to be significantly higher in autism [19, 21]. Plasma thiobarbituric acid-reacting substances (TBARS), which include MDA, were significantly higher in autism. These findings support the hypothesis that excessive ROS production and oxidative damages correlated with ASD [4]. Similarly, in our study, the MDA concentration was significantly higher in ALCL than in NALCL. This data revealed that autistic individuals have significantly higher oxidative stress than a normal person. However, the treatment with KH showed no significant difference in the MDA level between treated ALCL and the untreated group. We hypothesized that the significant effect could be accomplished with the adjustment of incubation time and doses of KH.

Other than lipids, oxidative damage occurs on nucleic acids which caused destructions on DNA strands such as breaks, protein crosslinking, and mutations [4]. Our data disclosed a significantly more severe DNA damage of Grades 2 and 3, indicating higher levels of DNA damage occurring in the autism cells than the normal cells. These results were congruent with the previous findings that reported the incidence of DNA oxidation damage and a deficit in antioxidant capacity in the plasma of ASD children compared to nonaffected siblings [22]. Interestingly, KH treatment successfully decreased the DNA damage (Grade 2) in ALCL after 24 h of incubation time.

Studies showed that manuka honey (MH) treatment protects macrophages induced with LPS (lipopolysaccharides) from oxidative damages [23]. ROS and nitrite production were inhibited; inflammatory markers were suppressed; proteins, lipids, and DNA of the cells were preserved; and antioxidant enzyme activities were restored in the macrophages [23]. Other data on MH also reported that supplementation with MH reduced DNA damage and MDA in the liver of young and middle-aged rats [12]. However, up to the present, no report was found concerning the effect of honey treatment especially stingless bee honey (KH) on the autism cells pertaining to oxidative stress and DNA damage.

*Gelam* honey (GH) is also reported to modulate endogenous antioxidant activities in rats [24]. GH increased the erythrocyte CAT and cardiac SOD activities in young and aged groups. DNA damage and MDA level, which increased in the aged rats, were reduced with GH supplementation [24]. Several studies on different types of honey treatment revealed the protective effects of honey on cisplatin- (CP-) induced oxidative damages. Oxidative stress markers (MDA and iNOS) were reduced, while antioxidant enzymes (SOD, GSH, and CAT) were increased with honey treatment on CP-induced oxidative stress [25].

Oxidative stress, which occurs from the cumulative impact of multifactor, can lead to neuronal damage and possibly play a critical role in the pathogenesis of ASD. A significantly lower concentration of GPx and SOD in the ASD group may indicate a reduced capacity of H_2_O_2_ neutralization and another ROS. This condition subsequently causes severe damage to the macromolecules. Reduced enzyme activities could result from (1) deficient production and/or (2) excessive consumption during the process to neutralize free radicals. Meanwhile, the greater oxidative damage occurring in autism is most likely due to (1) excessive oxidative stress and/or (2) the failure of restoring the endogenous antioxidant enzyme activity in response to stress. These collective impacts will probably contribute to the pathogenesis of autism.

Treatment with KH on autism cells effectively reduced the oxidative stress by modulating the increment of the endogenous enzymes, SOD and GPx activities, while reducing the oxidative stress and damages indicated by the decline of Grade 2 DNA damage. The increased endogenous enzyme activities exhibited with KH treatment could be due to the restoring effects of antioxidant enzymes in autism cells to neutralize H_2_O_2_ and accumulated ROS, thus protecting the cells from further oxidative damages to molecules.

## 5. Conclusions

KH, an antioxidant-rich natural product, has a high potential in alleviating oxidative stress, reducing the oxidative damage, and may help to improve the symptoms and problems of ASD.

## 6. Limitations and Future Directions

In vitro study is subject to certain limitations, and one of them is the inability to assess behavior and the variations in ASD characteristics. Another technical limitation is the limited number of cell lines used. These drawbacks limit the study for nonpersonalized treatment.

Significant findings from this study will be a reasonable justification for expanding the work to elucidate the protective effects of KH on cells under the ROS-challenged environment. ROS generation and the possible involvement of mitochondria would be the impactful parameters to be tackled in the future.

## Figures and Tables

**Table 1:** Grades of DNA damage.

Grade of genotoxicity	DNA damage
Grade 0 (no damage)	<5%
Grade 1 (low-level damage)	5%–20%
Grade 2 (medium-level damage)	21%–40%
Grade 3 (high-level damage)	41%–95%
Grade 4 (total damage)	>95%

## Data Availability

All data underlying the findings of the study are attached in this manuscript.

## References

[1] Lord C., Brugha T. S., Charman T. (2020). Autism spectrum disorder. *Nature Reviews Disease Primers*.

[2] Tye C., Runicles A. K., Whitehouse A. J. O., Alvares G. A. (2019). Characterizing the interplay between autism spectrum disorder and comorbid medical conditions: an integrative review. *Frontiers in Psychiatry*.

[3] World Health Organization (2019). Autism spectrum disorders. https://www.who.int/news-room/fact-sheets/detail/autism-spectrum-disorders.

[4] Manivasagam T., Arunadevi S., Essa M. M., Essa M. M., Qoronfleh M. W. (2020). Role of oxidative stress and antioxidants in autism. *Personalized Food Intervention and Therapy for Autism Spectrum Disorder Management. Adv Neurobiol*.

[5] Dolske M. C., Spollen J., McKay S., Lancashire E., Tolbert L. (1993). A preliminary trial of ascorbic acid as supplemental therapy for autism. *Progress in Neuro-Psychopharmacology & Biological Psychiatry*.

[6] Abirami G. P. P., Radhakrishnan R. K., Johnson E., Essa M. M., Qoronfleh M. W. (2020). The regulation of reactive neuroblastosis, neuroplasticity, and nutraceuticals for effective management of autism spectrum disorder. *Personalized Food Intervention and Therapy for Autism Spectrum Disorder Management. Adv Neurobiol*.

[7] Lewoyehu M., Amare M. (2019). Comparative evaluation of analytical methods for determining the antioxidant activities of honey: a review. *Cogent Food & Agriculture*.

[8] Saeed M. A., Jayashankar M. (2019). Therapeutic properties of honey. *Journal of Global Biosciences*.

[9] Hassan H., Karim N. A. (2019). Honey as versatile remedy: a focus on selected honeys. *International Journal of Food and Nutritional Science*.

[10] Hassan H., Yasmin Anum M. Y., Karim N. A. (2019). Antioxidant properties of stingless bee honey and its effect on the viability of lymphoblastoid cell line. *Medicine & Health*.

[11] Ferreres F., Andrade P., Tomás-Barberán F. A. (1994). Flavonoids from Portuguese heather honey. *European Food Research and Technology*.

[12] Jubri Z., Rahim N. B., Aan G. J. (2013). Manuka honey protects middle-aged rats from oxidative damage. *Clinics (São Paulo, Brazil)*.

[13] Al-Gadani Y., El-Ansary A., Attas O., Al-Ayadhi L. (2009). Metabolic biomarkers related to oxidative stress and antioxidant status in Saudi autistic children. *Clinical Biochemistry*.

[14] Söğüt S., Zoroğlu S. S., Özyurt H. (2003). Changes in nitric oxide levels and antioxidant enzyme activities may have a role in the pathophysiological mechanisms involved in autism. *Clinica Chimica Acta*.

[15] Al-Ayadhi L. Y., Elamin N. E. (2013). Camel milk as a potential therapy as an antioxidant in autism spectrum disorder (ASD). *Evidence-based Complementary and Alternative Medicine*.

[16] Thomas N. O., Effiong O. O., Otu O. V., Smith J. (2016). Impact of vitamins C and E supplement on anti-oxidant enzymes (catalase, superoxide dismutase, and glutathione peroxidase) and lipid peroxidation product (malondialdehyde levels) in sickle subjects. *Tropical Journal of Medical Research*.

[17] Khalaj R., Hajizadeh M. A., Zare M. (2018). Hesperetin and it nanocrystals ameliorate social behavior deficits and oxido-inflammatory stress in rat model of autism. *International Journal of Developmental Neuroscience*.

[18] Mostafa G. A., El-Hadidi E. S., Hewedi D. H., Abdou M. M. (2010). Oxidative stress in Egyptian children with autism: relation to autoimmunity. *Journal of Neuroimmunology*.

[19] Meguid N. A., Dardir A. A., Abdel-Raouf E. R., Hashish A. (2011). Evaluation of oxidative stress in autism: defective antioxidant enzymes and increased lipid peroxidation. *Biological Trace Element Research*.

[20] Rose S., Melnyk S., Pavliv O. (2012). Evidence of oxidative damage and inflammation associated with low glutathione redox status in the autism brain. *Translational Psychiatry*.

[21] Chauhan A., Chauhan V., Brown W. T., Cohen I. (2004). Oxidative stress in autism: increased lipid peroxidation and reduced serum levels of ceruloplasmin and transferrin--the antioxidant proteins. *Life Sciences*.

[22] Markkanen E., Meyer U., Dianov G. L. (2016). DNA damage and repair in schizophrenia and autism: implications for cancer comorbidity and beyond. *International Journal of Molecular Sciences*.

[23] Gasparrini M., Afrin S., Forbes-Hernández T. Y. (2018). Protective effects of Manuka honey on LPS-treated RAW 264.7 macrophages. Part 2: control of oxidative stress induced damage, increase of antioxidant enzyme activities and attenuation of inflammation. *Food and Chemical Toxicology*.

[24] Sahhugi Z., Hasenan S. M., Jubri Z. (2014). Protective effects of gelam honey against oxidative damage in young and aged rats. *Oxidative Medicine and Cellular Longevity*.

[25] Ridzuan N. R. A., Rashid N. A., Othman F., Budin S. B., Hussan F., Teoh S. L. (2019). Protective role of natural products in cisplatin-induced nephrotoxicity. *Medicinal Chemistry*.

